# Socio-cultural and service delivery dimensions of maternal mortality in rural central India: a qualitative exploration using a human rights lens

**DOI:** 10.3402/gha.v8.24976

**Published:** 2015-04-01

**Authors:** Tej Ram Jat, Prakash R. Deo, Isabel Goicolea, Anna-Karin Hurtig, Miguel San Sebastian

**Affiliations:** 1 United Nations Population Fund, Bhopal, India; 2 Division of Epidemiology and Global Health, Department of Public Health and Clinical Medicine, Umeå University, Umeå, Sweden; 3 Swedish Research School for Global Health, Umeå University, Umeå, Sweden; 4 Umeå Centre for Gender Studies, Umeå University, Umeå, Sweden

**Keywords:** maternal health, maternal death, right to health, rights-based approach, India

## Abstract

**Background:**

Despite the avoidable nature of maternal mortality, unacceptably high numbers of maternal deaths occur in developing countries. Considering its preventability, maternal mortality is being increasingly recognised as a human rights issue. Integration of a human rights perspective in maternal health programmes could contribute positively in eliminating avertable maternal deaths. This study was conducted to explore socio-cultural and service delivery–related dimensions of maternal deaths in rural central India using a human rights lens.

**Design:**

Social autopsies were conducted for 22 maternal deaths during 2011 in Khargone district in central India. The data were analysed using thematic analysis. The factors associated with maternal deaths were classified by using the ‘three delays’ framework and were examined by using a human rights lens.

**Results:**

All 22 women tried to access medical assistance, but various factors delayed their access to appropriate care. The underestimation of the severity of complications by family members, gender inequity, and perceptions of low-quality delivery services delayed decisions to seek care. Transportation problems and care seeking at multiple facilities delayed reaching appropriate health facilities. Negligence by health staff and unavailability of blood and emergency obstetric care services delayed receiving adequate care after reaching a health facility.

**Conclusions:**

The study highlighted various socio-cultural and service delivery–related factors which are violating women's human rights and resulting in maternal deaths in rural central India. This study highlights that, despite the health system's conscious effort to improve maternal health, normative elements of a human rights approach to maternal health (i.e. availability, accessibility, acceptability, and quality of maternal health services) were not upheld. The data and analysis suggest that the deceased women and their relatives were unable to claim their entitlements and that the duty bearers were not successful in meeting their obligations. Based on the findings of our study, we conclude that to prevent maternal deaths, further concentrated efforts are required for better community education, women's empowerment, and health systems strengthening to provide appropriate and timely services, including emergency obstetric care, with good quality.

As the year 2015 begins, the debate on the likelihood of achieving the Millennium Development Goals (MDGs) is gaining momentum (1–5) . Recent reviews of the MDGs show that the progress in achieving some of the MDGs is satisfactory but the advancement towards achieving MDG goal 5 (MGD 5), ‘Improving maternal health’, is off-track (2–7) . The United Nations (UN) General Assembly in its 65th session in 2010 has expressed grave concerns over the slow progress being made in reducing maternal mortality and improving maternal and reproductive health (8). The latest global estimates show that during the year 2010, around 287,000 women died due to maternal causes and 10–15 million more women suffered annually from severe obstetric morbidity. With 56,000 maternal deaths, India accounted for 19% of the global burden of maternal deaths in 2010 (3). The maternal mortality ratio (MMR) in India between 2007 and 2009 was 212, which is far away from the MDG 5 target for India: 109 maternal deaths per 100,000 live births (9). Around two-thirds of maternal deaths in India are estimated to take place in 9 out of 28 states and 7 union territories in the country, namely Assam, Bihar, Chhattisgarh, Jharkhand, Madhya Pradesh, Odisha, Rajasthan, Uttarakhand, and Uttar Pradesh. The MMR of Madhya Pradesh state, which is situated in central India, was 310 maternal deaths per 100,000 live births in 2010 (10).

Maternal health is prominently appearing on policy agendas of the Government of India (GOI) and the state government in Madhya Pradesh (11–15) . However, the quality of care and the use of maternal health services in the state remain low (16–18) .

The first organised initiative for maternal health care delivery in India was taken in the nineteenth century wherein the British colonial government condemned the natives for the enormously high maternal mortality rate and started the Lady Dufferin Fund in 1885 for providing maternity services. This was followed by the ‘women medical service’ by the British colonial government in 1914, wherein the government accepted its responsibility to provide maternity services. However, the availability and use of these services remained limited (19). The Health Survey and Development Committee, popularly known as the Bhore Committee, presented a plan of National Health Service in 1946 for providing free health services, including maternal health services, with universal coverage to the entire population through a comprehensive state-run health service. After independence in 1947, hospitals were established at district and higher levels to provide referral services. The maternal and child health (MCH) services were included in the basic public health programmes. However, the health services in the country mainly focussed on immunisation and birth control until the early 1990s, and maternal health remained neglected (19, 20).

The GOI launched the Child Survival and Safe Motherhood Programme (CSSM) in 1992 with support from the World Bank and United Nations Children's Fund (UNICEF). It focussed on CSSM services such as prevention of anaemia, antenatal care, immunisation, early detection and treatment of obstetric complications, delivery by trained personnel, institutional deliveries, and setting up of First Referral Units (FRUs) and referrals for institutional deliveries (21). It made some improvements in the service delivery but failed in realising its goal of increasing the number of well-functioning childbirth facilities in the country (22–24) . Encouraged by the Programme of Action of the International Conference on Population and Development (ICPD-POA) of 1994, the GOI reoriented its health programmes towards a rights-based approach and launched the first phase of the Reproductive and Child Health (RCH) Programme in October 1997, which offered client-centred, demand-driven, quality maternal health and family planning services (25, 26). It made additions into the services offered by the earlier CSSM by including safe abortion services, establishing blood storage units, and providing additional nursing staff at primary health centres (PHCs) for round-the-clock maternal health services (26, 27). However, this programme also could not achieve its desired objectives of reducing maternal mortality (28).

The period from 2000 to 2005 experienced many developments at international, national, and subnational levels. The announcement of MDGs that have improving maternal health as one of the goals contributed to encouraging the country to prioritise maternal health on the policy agenda. Three major national policies were adopted by the GOI during this period – the National Population Policy 2000, the National Policy for Empowerment of Women 2001, and the National Health Policy 2002 – and all of them endorsed the GOI's target of reducing the MMR to 100 maternal deaths per 100,000 live births (7, 29–31) . The National Rural Health Mission (NRHM) was launched in 2005 with major objectives to reduce maternal and child mortality by bringing all health, family welfare, and allied sector programmes under one umbrella. The GOI increased the funding for health programmes substantially and gave high priority to restructuring the rural health care delivery system in the country (14).

As part of the NRHM, a scheme called Janani Express has been implemented since 2006 in which all pregnant women are provided free transportation from their homes to health facilities and for referral to higher facilities for delivery. The Janani Express is funded by the GOI under the NRHM. For this, ambulances of government hospitals are used, and private vehicles are also hired by the government on contract and attached to hospitals for transportation of pregnant women for delivery. The pickup, drop-off, and referral of pregnant women by these vehicles are done free of cost. Under the scheme Janani Suraksha Yojana, started in 2006, all women from rural and urban areas are provided cash incentives of INR 1,400 (US $24) and INR 1,000 (US $17), respectively, for institutional deliveries in government health facilities as well as in accredited private health facilities (32). The Annual Health Survey (AHS) 2010–2011 shows that 90.1% of mothers in Khargone district availed themselves of cash incentives for institutional deliveries under Janani Suraksha Yojana. The introduction of cash incentives for institutional deliveries and other initiatives under the NRHM have encouraged women to deliver in health facilities. The AHS 2010–2011 reported 73.9% institutional deliveries in Khargone district compared to less than 25% in 2005 before the introduction of the NRHM (10). However, research suggests that, despite significant increases in crude coverage, effective coverage remains limited (33).

Under the NRHM, Janani Shishu Suraksha Karyakram (the Mother and Child Protection Programme) also has been implemented in the state since 2011; under this programme, the women are entitled to services in government health institutions free of cost, namely delivery care, including caesarean sections, drugs, diagnostics, food, and treatment of sick newborns up to 30 days. They are also entitled to exemption from user charges, and are offered free drugs and consumables, free diagnostics, free diet during their stay in the health institutions (3 days in case of normal delivery, and 7 days in case of caesarean section), free provision of blood, free transport from home to health facilities, free transport between facilities in case of referral, and free drop-off from health facilities to home after delivery. These schemes and programmes are in place with additional financial provisions to make them operational. However, challenges and constraints at the implementation level may restrain the use of services. Some of these challenges may include a lack of human resources, especially the technical specialists; an unequal distribution of health facilities in urban and rural areas; ensuring a regular supply of drugs and consumables in health facilities; poor quality of referral services; and low competence levels of birth attendants at providing emergency obstetric care (27, 34, 35).

## Human rights approach to maternal health

According to the estimates of the World Health Organization (WHO), 88 to 98% of maternal deaths are preventable (36). Considering its preventability, maternal mortality is being increasingly recognised as a human rights issue (37–46) . The Human Rights Council of the United Nations, in its resolution adopted in 2009, recognised the unacceptably high global rate of maternal mortality and morbidity as a health, development, and human rights challenge and expressed that a human rights analysis and integration of a human rights perspective in international and national responses to maternal mortality and morbidity could contribute positively in eliminating preventable maternal mortality and morbidity (45).

The international covenants underscore the indivisibility of human rights. Several international covenants recognised the right to health and health care (47, 48). The Universal Declaration of Human Rights (UDHR) 1948 was a milestone declaration in establishing normative human rights frameworks. Article 25 of the UDHR established everyone's right to a standard of living adequate for health, including food, clothing, housing, medical care, and necessary social services, and asserted that motherhood and childhood are entitled to special care and assistance (47). Later, the convention on the elimination of all forms of discrimination against woman clearly recognised the right to reproductive and maternal health (49).

Several organisations and scholars have also conducted holistic assessments of maternal mortality and brought more clarity on human rights in the context of maternal health. The major contributions in this regard have been from the WHO, the UN Human Rights Council, Amnesty International, Paul Hunt, and UN Special Rapporteur on Right to Health, among others (45, 46, 50–54) . Critics of the right to health raise concerns over how to ensure achieving this goal as they believe that this right is an abstract right which is not justiciable. It is also argued that the right to health, being a welfare right, contrasts liberty rights (55).

A rights-based approach to maternal health identifies the centrality of a just and effective health system ensuring that maternal, reproductive, and sexual health services, goods, and information are available, accessible (physically accessible, financially affordable, and based on equality and non-discrimination), acceptable (ethically and culturally), and of good quality (56). It aims at empowering women to claim their rights with active participation in decision making and also highlights the importance of social determinants of maternal health. It also calls for establishing and effectively implementing mechanisms for ensuring accountability of all actors concerned with maternal health and women's reproductive and sexual health (51, 52, 56).

According to the recent technical guidance issued by the UN Human Rights Council, a human rights–based approach to maternal health identifies rights holders and their entitlements, and corresponding duty bearers and their obligations, and promotes strengthening the capacities of both rights holders to make their claims and duty bearers to meet their obligations (57). The primary obligations of the state parties regarding the right to health are to respect (states must not interfere with the right to health by adopting discriminatory policies), protect (states must ensure that non-state actors do not infringe on enjoyment of the right to health), and fulfil (by taking positive steps for realising the right to health through policy, legal, budgetary, and administrative measures). For meeting these obligations, states must take actions to ensure women's access to maternal health care along with other relevant reproductive health care services. States must increase resource allocation for maternal and reproductive health care services, develop and implement policies and programmes to ensure availability and access to these services by women without discrimination, improve transportation to the existing services, raise knowledge and awareness in the communities regarding complications and services, and address the social, cultural, and economic factors that constrain women's access to these services (46, 56, 57).

A rights-based approach to maternal health calls for a critical investigation of the factors associated with maternal deaths from a human rights perspective. The present study is part of a larger study exploring maternal health in Madhya Pradesh state, India. The objective of this study was to explore socio-cultural and service delivery–related dimensions of maternal deaths in rural central India using a human rights lens. The human rights approach has been previously used for exploring maternal mortality in various settings (46, 52–54, 58, 59), and this study builds on them. The key strength of this approach is that it provides a framework for examining the issues around maternal mortality in a holistic manner in the context of the rights of the right holders and the obligations of the states and health systems as duty bearers. However, this study is unique in the sense that we have combined the three delays model with a human rights framework for exploring the factors associated with maternal deaths in rural central India.

## Materials and methods

### Study setting

**Fig. 1:**
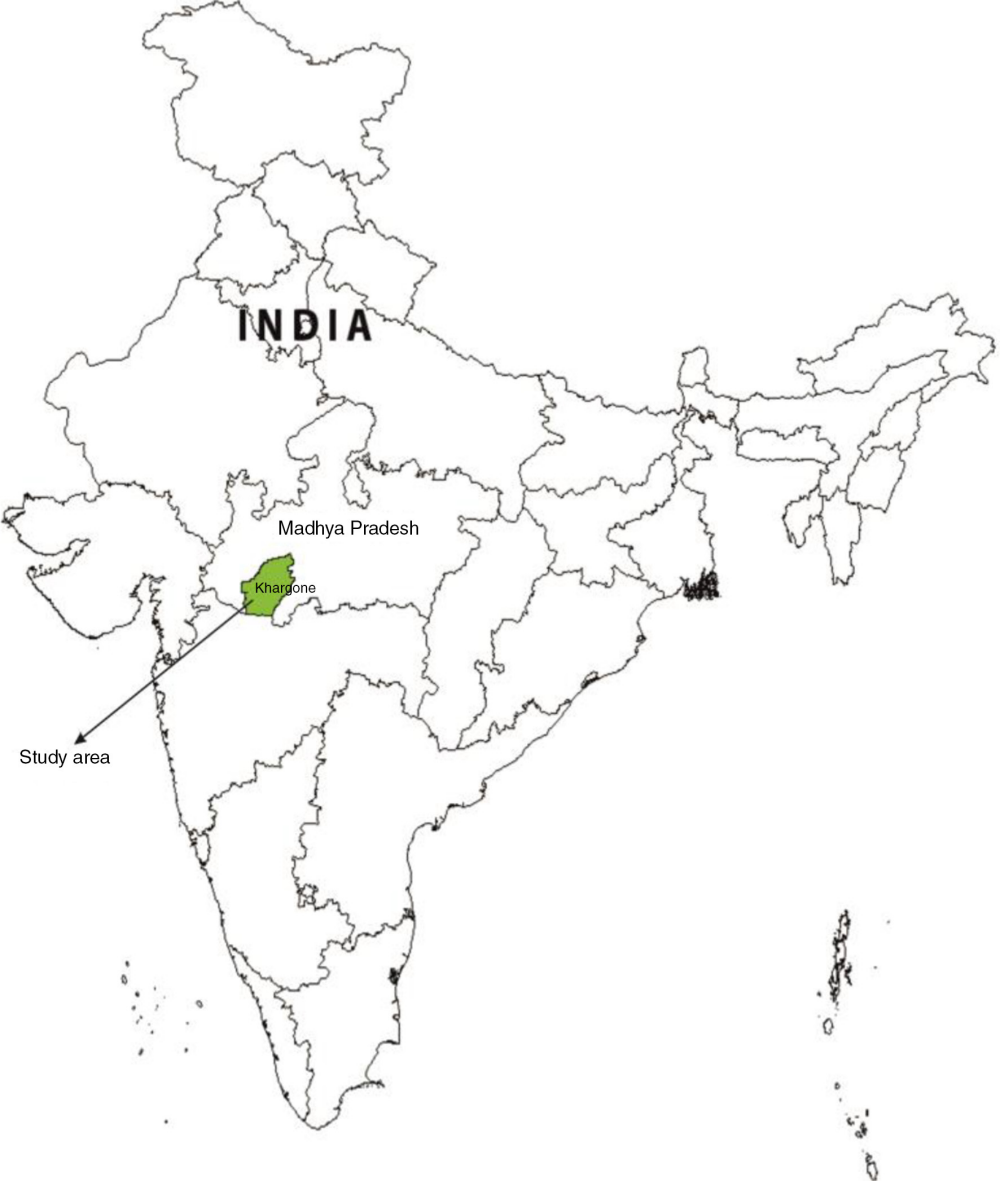
Map of India showing the study area.

The Khargone district was selected for this research due to its mix of tribal and other population groups in rural areas, the proportion of maternal deaths (Khargone, 12.2%; and Madhya Pradesh, 12.6%) in all deaths of women in the reproductive age group, and the status of health services similar to the situation in the state (10, 60, 61). This district is situated in the southwestern part of Madhya Pradesh state, which is located in central India (Fig. 1). It is surrounded by the Vindyachal and Satpura mountain ranges.

According to the Indian Census 2011, the district has a population of 1,872,413 with 953,433 males and 918,980 females. The district has 84% rural and 16% urban population. The population density of the district is 233 persons per square kilometre. The decadal growth rate of population in the district during 2001 to 2011 was 22.8%. The total literacy rate among the 7-year-olds and older is 64%. A huge gender gap exists in literacy rates in the district, as the male and female literacy rates are 74 and 53.7%, respectively (60). The third round of the District-Level Household and Facility Survey (2007–2008) reported 21.8% of girls getting married before the age of 18 years, which is the legal age for marriage (18). The same survey reported that only 64.5% of married women in the age group of 15 to 49 years use any modern method of family planning in the district.

The major sources of health care services in the district are government health facilities. As of March 2010, there were one district hospital, two civil (sub-district) hospitals, 10 community health centres, 54 PHCs, and 273 sub-health centres in the district (61). Normal delivery services are provided at some accredited sub-health centres. All PHCs and some community health centres are designated as basic emergency obstetric care (BEmOC) centres. The BEmOC centres provide parenteral administration of antibiotics and oxytocic drugs, provide anticonvulsants for pregnancy-induced hypertension, and perform manual removal of placenta, removal of retained products of conception, along with assisted vaginal deliveries. Selected community health centres and civil hospitals are designated as comprehensive emergency obstetric care (CEmOC) centres, and, along with the district hospital, these facilities are assigned to function as referral units for conducting complicated deliveries and provide CEmOC services. The CEmOC centres provide all basic emergency obstetric services and also perform caesarean sections and blood transfusions. The patients are referred to the medical college in Indore for tertiary care services. This medical college is around 120 km from the Khargone district headquarters.

The district has 123 medical doctors, including specialists. Out of them, 37 doctors are posted at the district hospital Khargone, 15 doctors are posted in two sub-district hospitals, and the rest are posted in community health centres and PHCs in rural areas of the district. The district has 367 staff nurses posted at the district hospital, community health centres, and PHCs in the district. The district also has 285 auxiliary nurse midwives posted at sub–health centres in the district. All of these service providers can perform normal vaginal deliveries, whereas the caesarean deliveries are performed by specialist doctors only.

### Study design

This research was designed as a qualitative study. Social autopsies were conducted for 22 cases of maternal deaths during the months from July to November 2011 in the Khargone district of Madhya Pradesh state (62–64) . Family members and relatives of the deceased women who witnessed the circumstances leading to death were interviewed using an adapted social autopsy tool for the study (63, 64). Verbal autopsy is used to determine the levels and causes of death among people who die outside of health facilities and/or without registration. With adaption, social autopsy has emerged as a methodological sub-field for qualitative interpretation of verbal autopsy data (34, 64–69) .

In this paragraph, we describe the adaptions made to the verbal autopsy tool for social autopsy purposes. We included both structured and open-ended questions in the questionnaire to bring in the qualitative dimension. Information on background characteristics of the deceased women and their families; history of any existing illnesses prior to pregnancy; pregnancy history; antenatal care; complications during pregnancy, abortion, labour, delivery, or the postpartum period; injury; and care seeking for obstetric complications that led to death were obtained through structured questions. In addition, open-ended questions were asked to collect the open history, wherein the respondents were prompted to narrate all the facts related to the complications that led to death and the care seeking for those complications. This qualitative part of the tool was an attempt to elicit the perceptions of respondents about the factors and conditions surrounding the maternal deaths. The respondents were encouraged, in this part of the interview, to reconstruct and describe the most relevant aspects of the circumstances that led to maternal deaths. The interviews were conducted from 2 weeks to up to 1 year after death. The questionnaire was pre-tested in one of the neighbouring districts in the state prior to its use for data collection in the present study. Based on the pre-testing, we modified some of the existing questions and added some new questions in the tool based on the experience of the field testing to address the overall research questions.

The cases of maternal deaths were purposively selected using the government records and community informants to cover a large spectrum of scenarios and circumstances associated with maternal deaths. Single interviews were conducted with eight husbands, four mothers-in-law, one father-in-law, and one sister-in-law of deceased women. In eight interviews, more than one respondent was present. In five interviews, husbands and mothers-in-law responded. In one interview, the husband and husband's younger brother were present; and, in one interview, the husband and brother-in-law of the deceased woman jointly responded. The mother-in-law, father-in-law, and husband of a deceased woman jointly responded in one interview.

A total of 22 maternal deaths were studied in this study. The respondents were selected based on their presence with the deceased women during the development of complications, transportation to health facility, treatment, referral, and death. We first approached the respondents in person, explained the objectives of the study to them, and fixed an appointment with them for an interview based on their availability and convenience. The power dynamics of familial relationships may have influenced the data in the form of dominant responses from the elder family members, as we included more than one relative or family member as respondents in the interviews. However, we tried to create such an environment during the interviews in which all respondents provided their views and no participant dominated the responses. Along with the first author, a physician and a social worker were involved in data collection. The interviews were conducted in the houses of the deceased women. We did not pre-decide the number of cases of maternal deaths.

A total of 22 cases of maternal deaths were followed up, and data collection stopped when we felt that saturation was reached (new information regarding the research question stopped emerging from the interviews). All interviews lasted between 75 and 120 min. In order to elucidate or elaborate some responses, follow-up interviews and phone calls were made in some cases. We examined a sub-set of narratives by discussing them with the informants to check the accuracy and ensure the quality of data. To protect the informants from harm in terms of the distressing nature of interviews, we took informed consent, assured them about anonymity, and told them that they were free to withdraw at any time or refuse to answer any specific question; we also arranged debriefing of informants after conducting interviews. The standard WHO definition of maternal death was used for this study (70).

### Data analysis

All interviews were conducted in the Hindi language and were tape recorded. The recordings of the interviews were transcribed verbatim and translated into English. To maintain anonymity, the verbal autopsies of individual cases of maternal deaths were assigned code numbers from 1 to 22. The data were analysed by using thematic analysis (71, 72). Thorough familiarity with the responses was gained by reading and re-reading all of the transcripts several times. Open Code 3.4 software was used to manage the process of coding and analysing the data (73). The codes from the different transcripts were reviewed while maintaining the principle of constant comparison. This principle empowers the researchers to discover and generate codes, categories, and concepts that help make a complex world more understandable and transparent. In this process, the researchers compare incident to incident, then, as a category or its property emerges, they compare the concept to the next incident (74). We used the principle of constant comparison in our analysis due to the complex nature of the phenomena under study.

**Fig. 2:**
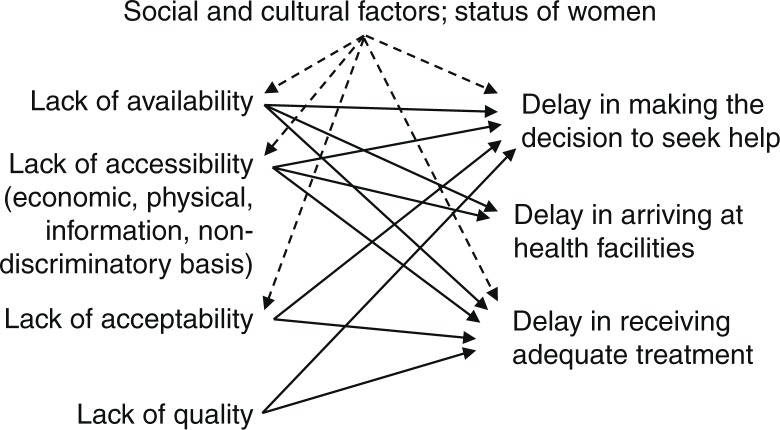
The ‘three delays’ model and lack of elements of a human rights approach to maternal health (59).

We applied the ‘three delays’ framework of Thaddeus and Maine to organise the findings of this study (75). This framework is based on the basic premise that many maternal deaths occur in developing countries because women experiencing obstetric complications do not get adequate care in time. According to the framework, this lack of care can be due to, first, delay in making the decision to seek care when obstetric complications develop; second, delay in reaching an appropriate health facility once the decision to seek care has been made; or, third, delay in receiving proper and adequate care after reaching a health facility (75). This framework has been used by researchers in different countries to examine the factors associated with maternal and neonatal deaths (67, 76). This framework was very useful as a classic conceptualisation of delayed treatment in obstetric emergencies. Maternal health services lacking the essential elements of a rights-based approach to maternal health contribute to the occurrence of the above three delays. The factors emerging from the study influencing the three delays are further examined in the ‘Discussion’ section regarding their linkages with the essential elements of a human rights approach based on the interactions illustrated in Fig. 2 (56, 59).

### Ethics

Ethical approval for the study proposal was obtained from the Ethics Committee of the Bhopal Regional Technical Centre of the Family Planning Association of India. Approval from the concerned government authorities was also obtained. Informed verbal consent was taken from each respondent after explaining the study objectives and assuring them of the confidentiality of their identity. Permission from all respondents was taken for recording the interviews. All the interviews were conducted at least 2 weeks after the death, which is the culturally prescribed mourning period in the area. The respondents were also told that they were free to decide not to answer any questions and were free to stop the interview and withdraw from the study at any time, and this decision would not affect the respondent or his or her family in any way. All the respondents were also asked whether they could be contacted again for any clarification if required, and all of them agreed to it.

## Results

A total of 22 cases of maternal deaths were studied. The average age of the deceased women was 26.3 years, ranging from 19 to 40 years (SD=5.4). A total of 20 out of 22 deceased women were between 19 and 34 years of age. Six of the 22 women were illiterate, seven studied for up to 5 years, and nine women studied for 6–11 years. None of them received higher education. Half of the deceased women were from scheduled tribes and scheduled castes, and the other half were from general castes and other backward classes which are socio-economically better off than the first half. More than half (12) of the women died at health facilities, whereas nine died en route to health facilities. One death occurred at home. Around one-third (seven) of the deceased women in the study were primiparous, and the other 15 were multiparous. The respondents reported that all of the deceased women were registered for antenatal check-ups (ANCs), whereas 18 and 4 women received three and two ANCs, respectively. The interviews also revealed that nine and 13 women received one and two tetanus toxoid injections, respectively, during the pregnancy period. Background characteristics of the cases are presented in Table 1.

**Table 1:** Profile of deceased women (maternal deaths)

Background characteristics	Number (*N=*22)
Age (years)	
19–26	10
27–34	10
35 and older	2
Education	
Illiterate	6
1–5 years	7
6–11 years	9
Caste^a^	
Scheduled tribe	2
Scheduled caste	9
Others	11
Place of death	
Home	1
Health facility	12
En route to health facility	9
Obstetric history	
Primiparous	7
Multiparous	15

a Scheduled castes (SCs) and scheduled tribes (STs) are officially recognised by the Government of India as socially and economically backward castes and tribes that are in need of special protection from exploitation and injustice. Other backward class (OBC) is the group of castes that are socially and educationally backward but enjoy higher status than SCs and STs in the caste hierarchy; the government is enjoined to ensure their social and educational development. Any person not belonging to any of these three groups is by default a member of a group classified as a general/forward caste (i.e. having the highest status in the caste hierarchy), and he or she is not eligible for any affirmative discriminatory support by the government.

The data from social autopsy interviews showed that the major causes of maternal death were haemorrhage in nine cases, sepsis in three cases, eclampsia in two cases, obstructed labour in one case, abortion in one case, severe anaemia in two cases, and other conditions in four cases. All 22 women tried to access medical assistance for occurrence of obstetric complications. Because the focus of this article is on the socio-cultural and service delivery dimensions of maternal deaths, detailed descriptions of the results related to medical causes of maternal deaths are not provided here. The findings of the qualitative part of this study are presented in the remainder of this section using the ‘three delays’ framework. Relevant quotations from the interviews are provided in italics.

### Delay in deciding to seek care

This delay was determined by the interval between the occurrence of an obstetric complication and when the decision to seek care was made. The analysis showed that half of the deceased women in the study experienced delay in seeking medical assistance after becoming aware of the obstetric complications.

The respondents perceived that they underestimated the severity of complications based on the experiences of previous deliveries of the deceased women and other women in their families, and they considered the problems to be normal without understanding their severity. This underestimation led to delay in deciding to seek medical assistance for childbirth which contributed to maternal deaths. The mother-in-law of a deceased woman reported that:

She was fine so we thought that there was no need to take her to any health facility. It was her second pregnancy and the first delivery happened without any problem. She started complaining about pain in the day at 12.30 PM but we waited at home in the hope of a normal delivery. When she did not deliver till evening and the whole night passed and the pains increased, we took her to the primary health centre in the next day early morning at 5.30 AM. (Case 9)

Some respondents [mothers-in-law] observed that the lack of participation of the parturient in decision making regarding accessing maternal health care also deterred and delayed the women from receiving timely delivery services.

My daughter in law was pregnant for the first time. As per our family tradition she was taken to her paternal home by her parents in the seventh month of her pregnancy. In the ninth month, one afternoon she started to have abdominal pain that continued for the day and night. She requested to be taken to a hospital but her parents wanted to take our consent before taking her to hospital as in our families consents of in-laws and husband of married daughter are considered to be a must for any important decision about a married daughter's health. For this they sent someone to our village in the next day morning, and I and my husband went with him. Then we took her to a government hospital [community health centre]. We reached there by the evening. The doctor checked her and said that the foetus had already died. Doctor conducted delivery in which she delivered a stillborn baby and within a few minutes after it she started a lot of bleeding and she died. (Case 16)

In some cases, the families of the deceased women perceived the quality of delivery care provided in government hospitals as poor based on the previous negative experiences of deceased women and other women in their families. Relatives of some deceased women were hesitant to take the pregnant women to the government hospital even if they were aware of the free availability of delivery care in these hospitals. The husband of a deceased woman narrated:

This was the third pregnancy of my wife. Our second baby was delivered two years back in a government hospital. I took her to the government hospital where she was admitted. The beds were not enough in the hospital for all pregnant women admitted in that hospital therefore she was asked to sleep in the corridor. […] Doctors were there but they did not come to examine my wife. Her examination was done by a nurse. My wife kept complaining about pain but nobody paid attention to her situation. […] Because of this kind of experiences we decided this time [for the third pregnancy] not to go to hospital for a normal delivery and waited for delivery to happen at home. But when she did not deliver and had severe pains we had to take her to the government hospital. (Case 4)

### Delay in reaching an appropriate health facility

This theme reflects the factors associated with the delay in reaching health facilities once the decision was made to seek care. This delay was determined by the difference of time between the decision to seek care and reaching the health care facility (where the woman received obstetric care). In some cases, significantly less time was taken in reaching the health facility, but there was a first delay that already complicated the cases. Twenty-one of the 22 women got delayed in reaching an appropriate health facility for obstetric care due to various obstacles. This delay reduced the likelihood of their survival. Transportation issues become very important in acute and time-limited conditions such as haemorrhage and other direct causes of maternal deaths. One woman died on her way to the first health facility, 12 died in health facilities, and 8 women died during the referral from one health facility to another health facility.

The respondents mentioned various problems related to transportation. Despite the government's provision of free transportation to bring women from their homes to health facilities and referral to higher facilities, vehicles were not available on some occasions. Women also faced transportation problems even after reaching the first health facility. Their referrals were delayed due to either the unavailability of a vehicle or the reluctance of health care staff to provide free transportation. A father-in-law narrated:

She had no problems during the pregnancy and when the pregnancy was of eight months, she started having pains. We took her to the district hospital by a private vehicle. The doctors at district hospital told us that her condition was serious as she was severely anemic. Three bottles of blood were arranged by us and given to her but her condition did not improve. We were told by the doctors to take her to Indore medical college. […] We took her to the medical college by a private vehicle as no government vehicle was made available from the hospital. We were told that the ambulance was not available. […] It took us around one hour to arrange a private vehicle. On the way she delivered a dead child in the vehicle. There was also excessive bleeding. […] Later just after some time she passed away in the vehicle. (Case 13)

Problems in proper implementation of the free transportation scheme, such as a lack of fuel for the Janani Express vehicle due to a lack of proper management and supervision, were reported by the respondents, and these delayed women from reaching health facilities for appropriate delivery care. The husband of a deceased woman shared:

When my wife told us about her pains and breathlessness, we decided to take her to the government hospital. We made a call on the telephone numbers of Janani Express but we were told that the vehicle was running out of fuel. Then we looked for a four wheeler but when we did not find a vehicle we took her to the community health centre on a motor cycle. (Case 21)

Lack of awareness regarding the availability of free transportation was also mentioned by a few respondents.

We were not aware about the free vehicle facility provided by the government for delivery at the health centre. Therefore, we tried to arrange a private vehicle from the nearby village, which took me two and half hours. Then we started to take her to the government hospital. But on our way to the hospital, she had very strong pain and bleeding. Then she died in the vehicle. (Case 12)

Care seeking at multiple facilities or multiple referrals by health care providers also contributed in delaying access to timely and appropriate care. Thirteen of the 22 women accessed services from more than two health facilities, because either they were referred from one facility to another or their families took them for care to more than two health facilities. Some participants mentioned that they themselves had to take the deceased women to more than one facility as they were not satisfied with the care provided in the first facility. The husband of a deceased woman narrated that:

The doctor admitted her but did not provide any treatment for three hours and she constantly kept complaining about severe pain and also started bleeding heavily. Then we [the family members] decided to take her to another hospital for services. (Case 18)

Some respondents also mentioned multiple referrals by the service providers. The mother-in-law of a deceased woman narrated:

One day she suddenly started to feel pains and we took her to a health centre [primary health centre] from where she was referred to another health center [community health center]. This transportation was done by the Janani Express vehicle. In that health centre she was told that she had high blood pressure and she was referred to the District Hospital. By now the pains had increased and she was having perplexity and uneasiness. In the district hospital, without telling any reason they again referred her to the medical college. (Case 3)

### Delay in receiving proper and adequate care after reaching a health facility

This delay was determined by the time taken to initiate definitive obstetric care once the women reached the health facility. Analysis of interview data revealed that 13 out of 22 deceased women experienced this delay.

The respondents mentioned that the health staff showed negligence in immediately providing proper care, in treating obstetric complications, and during referrals. They also reported that some women were referred to other facilities unnecessarily, even without telling any reason to the family members who were accompanying the deceased women. The husband of a deceased woman narrated:

She started having pains and we took her to a health centre [community health center], where she delivered a baby by normal delivery. Four days later she complained of having foul smelling discharge from vagina along with blood. She also developed high fever. We took her again to the same health centre and from there they [Doctor and Nurses] referred her to the District Hospital. She was not treated well there and she was referred to the medical college without giving her any treatment. She died on the way to the medical college …. (Case 6)

Some respondents also reported that the deceased women could not get appropriate obstetric care services, including blood transfusion in community health centres and the district hospital, to manage the complications in a timely manner, even after reaching the health facilities that are designated as comprehensive EmOC centres. The husband of a deceased woman reported:

She was in her eighth month of pregnancy. She started having pains so we took her to the nearest community health centre. We were told that there were no facilities to manage her problem in that health centre and they referred her to the district hospital. We arrived in the district hospital at night where she was advised [to have] surgery. Doctor told us that there was no blood available in the hospital, and he asked us to arrange three bottles of blood but we could arrange only two bottles. After the surgery the doctor told us that she died due to loss of blood. (Case 7)

In another case, the husband of a deceased woman explained:

After home delivery she started bleeding and the placenta was not delivered so we took her to the district hospital one and half hour after delivery. By that time she had become unconscious and was continuously bleeding. When we reached the district hospital we were told to immediately take her to the medical college. We started to take her to the medical college after 30 minutes but she died en route to the medical college. (Case 2)

## Discussion

Health systems are not merely mechanical structures to deliver technical interventions; rather, they are considered to be core social institutions for respecting human rights (46, 77). Effective health systems functioning based on the principles of justice and equity have a central place in a human rights approach to maternal health (56). Following the emergence of maternal health on the priority policy agenda, the GOI and the state government in Madhya Pradesh are making several concentrated efforts to strengthen the health system for providing maternal health services and to increase the demand for these services (14, 15, 26). Some of these efforts have been successful to some extent, by increasing institutional deliveries in the state from 28% in 2002–2004 to more than 76% during 2007–2009 (10). However, the research evidence shows that crude coverage has increased, but effective coverage remained limited as the increased institutional deliveries could not proportionally accelerate the reduction in maternal mortality (33). The efforts made by the government in this regard are not sufficient to address the issues related to maternal mortality in Madhya Pradesh, as the implementation of these programmes and addressing the socio-cultural and human rights dimensions need further attention. The accounts of events surrounding the deaths of deceased women narrated by their relatives make it clear that the duty bearers could not meet their obligations in respecting, protecting, and fulfilling the women's rights in the context of maternal health; in creating a conducive environment for women to enjoy their entitlements relating to goods, services, and information; in empowering women to make decisions; and in improving other social determinants of maternal health.

The factors influencing the delays emerging from our study and their linkages with the elements of a human rights approach to maternal health are illustrated in Table 2.

**Table 2:** Elements of a human rights approach to maternal health, and factors influencing the delays

Elements of a human rights approach to maternal health	Factors influencing the delays	Delays^a^
Availability	Lack of blood in health care facilities Unavailability of free transportation Lack of fuel for *Janani Suraksha* vehicles	2 and 3
Accessibility (information, economic, physical, and non-discrimination)	Inaccessibility of information among relatives on the warning signs for obstetric complications Negative perceptions of families regarding delivery services Lack of proper information regarding free transportation for institutional deliveries Gender inequity hindering women's decision-making power	1, 2, and 3
Acceptability	Negative perceptions of the families regarding delivery services	1
Quality	Negligence by health staff in providing care Lack of blood and other emergency obstetric care services Reluctance of service providers to provide free transportation for referral	1 and 3

a Delay 1: delay in making the decision to seek care; Delay 2: delay in reaching an appropriate health facility for care; and Delay 3: delay in receiving adequate care after reaching a health facility.

### Availability

A human rights approach to maternal health acknowledges that health facilities, goods and services must be available in sufficient quantity (56). Research in different parts of the world has established that providing basic and comprehensive EmOC services in case of obstetric complications can avert a large number of maternal deaths and lack of these services contributes hugely in higher levels of maternal mortality in several developing countries (75, 78). The UN guidelines recommend at least four basic EmOC and one comprehensive EmOC facilities for a population of 500,000 (79). The study district has a population of 1.8 million and it has 24 and three facilities designated as basic and comprehensive EmOC, respectively. However, many of these facilities are not fully functional; therefore, all women included in the study could not receive appropriate obstetric care within the district. They had to seek care at multiple facilities or they were referred to other facilities out of the district without providing them adequate care. The issue of unavailability of blood also emerged in our study. These findings are consistent with the results of another study conducted in the Barwani district in Madhya Pradesh that highlighted the issue of unnecessary referrals because of the unavailability of required services or indifferent attitude of the service providers (80).

The availability of an appropriate transportation system for referral linkages assumes paramount importance in the cases of obstetric complications. The problem of unavailability of transportation was faced by the deceased women. Despite the government's scheme *Janani Express Yojana* to provide free transportation to women for institutional delivery (14, 32), women died on the way to health care facilities. The main highlighted problems were the unavailability of vehicles and lack of fuel. The provision of free transportation to women for delivery care exists but due to the problems in its implementation women cannot use it when they need it. Negative reinforcement between the transport issues and the multiple referrals was also observed. This situation calls for an in-depth enquiry into the functioning of this scheme and for further corrective actions. The implementation of free transportation scheme should be strengthened with more rigorous monitoring and ensuring accountability of the concerned people. The most important implication for practice is strengthening the implementation of the scheme by improving monitoring and supervision which provides free transportation for delivery care along with reducing unnecessary referrals from one facility to other.

### Accessibility

The accessibility element of a human rights approach to maternal health includes four interrelated dimensions namely: information, economic, physical and accessibility based on non-discrimination. Information accessibility includes primarily awareness of the warning signs for obstetric complications and about the maternal health services. Timely identification of complications and understanding their severity are crucial steps in deciding to seek medical care and avoiding maternal deaths (delay 1). Related factors such as underestimation of complication symptoms by family members, lack of women's autonomy and negative perceptions regarding delivery services delayed the decision to access medical assistance in cases of obstetric complication, which resulted in maternal deaths. These results are similar to those reported by another study (81) conducted in the Satna district of the state, which reported that in 21.4% of the cases families showed lack of knowledge for recognising the symptoms of the complications. Despite the provision of free transportation for institutional deliveries by the government, lack of proper information about the free transportation service also emerged from our study as a factor delaying the access to obstetric care.

The women's subordinate status is a key social determinant of poor maternal, sexual, and reproductive health, particularly in India (58, 82, 83). Our findings indicated that the gender inequity hindering women's decision-making power led to maternal deaths. Women in the local society are traditionally not given autonomy to make important decisions about themselves including their health and delivery care, as well as their family-related matters. These decisions are made in a complex web of power relations. Women face gender-based discrimination and they are given lower status in the families and the society. Decisions related to seeking medical assistance for delivery are generally taken by husbands or other elders in the families. The elder women in central Indian society have more respect in comparison with younger women. However, the most important and critical decisions are generally taken by the elder male members of the families. Many pregnant women are taken to their parental homes by their parents for care taking during pregnancy, delivery, and post-natal period. But parents make all important decisions about their married daughters with consent from husbands and in-laws of the daughters. The third round of the National Family Health Survey (2005–2006) reported that only 43.3% currently married rural women in Madhya Pradesh usually participate in making household decisions, such as making household purchases for daily household needs, visiting their own families or relatives and seeking care for their own health. Our findings regarding women's low status in decision-making are consistent with a previous study conducted in the state (81).

Economic accessibility in terms of affordability is a very crucial element of a human rights approach to maternal health. Other studies in Madhya Pradesh during the period 2002–2003, and in other Indian states from 2002–2003 to 2006–2008 (34, 65, 81), reported financial constraints as one of the factors associated with the delay in deciding to seek delivery care, but this issue did not appear in our study. This might be due to reasons such as delivery care being provided by the government health facilities free of cost and the provision of cash incentives under Janani Suraksha Yojana (14, 32), or it may be due to an increased flow of cash in the economy resulting from the implementation of several flagship government schemes and economic reforms. This issue needs to be further investigated.

### Acceptability

Perceptions of users regarding the maternal health care services tremendously influence the use of these services (59, 84). Our results showed that negative perceptions of the families regarding delivery services constrained the use of appropriate obstetric services by the women when they needed them the most. This finding is aligned with the results of various studies in the state and in other parts of the country (17, 80, 85) that highlighted the poorly provided maternal health services as barriers to accessing maternal health services, especially EmOC.

### Quality

An effective health system with high-quality EmOC services is an essential element of a human rights approach to maternal health. According to the Economic, Social, and Cultural Rights Committee of the United Nations, ‘health facilities, goods and services must also be scientifically and medically appropriate and of good quality. This requires, *inter alia*, skilled medical personnel, scientifically approved and unexpired drugs and hospital equipment’ (56). Lack of human resources, equipment, and drugs and/or the insensitive attitude of the health care providers results in poor quality of delivery services, which may result in maternal deaths. The factors behind the poor-quality delivery services that emerged from this study were negligence by health staff in providing care and lack of blood and other EmOC services. Negligence committed by and insensitive attitudes of health care providers constrained women's freedom to exercise their reproductive and sexual rights related to maternal health as they were not able to claim their lawful entitlements. The probable reasons behind the insensitive behaviour of the service providers may be overburdened health facilities and shortages of human resources, resulting in lack of proper attention to the patients. This aspect of the failure of the health system as a duty bearer needs to be further investigated. The lack of blood has also emerged from this study as one of the key problems. The state government has established blood banks at the district hospital level and blood storage units at other facilities providing CEmOC. Sometimes, there is lack of blood due to non-availability of donors, non-availability of blood from the required blood group, and sometimes mismanagement at the level of the blood banks or blood storage units. Proper management of blood banks and blood storage units with more training and supportive supervision, and encouraging more donors, may help in addressing the problem of lack of blood.

The results of this study regarding the quality dimension of obstetric care align with those of previous studies in Madhya Pradesh and other Indian states (34, 35, 80). Some of the issues influencing the quality of maternal health services are also linked with other dimensions, such as the availability of human resources, drugs, and equipment, as well as the acceptability of services.

### Combined effects

The combined effects of the issues surrounding different elements of the human rights framework are noteworthy, as the problems around these issues interconnect. Table 2 shows that transport issues are observed in three of the four elements of the human rights framework, and these issues contribute to all three delays. The unavailability, lack of information, and reluctance of staff to provide transport, along with the second issue of referrals, indicate that despite efforts to provide accessible care, stimulating demand without fully strengthening the supply of services results in the unintended consequences of overburdened facilities and providers who may be unable and unwilling to care for women with critical complications. This affects the accessibility as well as the quality of care. This poor accessibility combined with poor quality negatively impact the acceptability of treatment, as the women and their families have to make critical decisions about attending poor-quality services. The interconnectivity reinforces and maintains the poor availability, accessibility, acceptability, and quality of services observed in the three phases.

The use of a human rights lens provided us with a very useful framework for a comprehensive exploration of maternal mortality in rural areas of a country committed to significantly reducing the MMR. The findings of this study make an important contribution to our understanding of the socio-cultural and service delivery factors associated with maternal deaths in central India. These findings will be useful for policy makers, programme managers, and researchers to better grasp and avert preventable maternal deaths in India as well as in other countries with similar settings.

### Methodological considerations

This study has some limitations which need to be mentioned. The cases of maternal deaths for the study were selected purposively to cover a large spectrum of scenarios, and we tried to detail the analytical process and contextualise the results. Selection bias may have occurred through the way we identified deaths by going through facilities and community informants. Some maternal deaths may have been missed in this process that may have particular characteristics related to social exclusion from access to health and health care services. We may have missed these characteristics in our analysis. The findings presented in this article are based on self-reporting by the relatives of the selected deceased women, who may have provided some inaccurate or incomplete information. It would have been useful to collect information from other stakeholders, such as maternal health care providers; but, due to limited resources for conducting the study, we decided to focus on the perceptions of relatives of deceased women and how they perceived the factors contributing to maternal deaths. This was because our aim was to hear the voices of those affected, since their experiences regarding the delivery care services are worth exploring, because even if they might not truly reflect the situation, they definitely influence their care-seeking behaviour. Since the interviews were conducted from 2 weeks after death to up to 1 year, recall bias may have occurred in this study. However, in order to avoid this bias, we included more than one respondent in interviews and gave them enough time to recall the events and information correctly. Based on our experiences of conducting this study, we believe that perhaps following up the cases of maternal deaths using a social autopsy approach might be a further route to gain acute perspectives on the lived experience of care seeking in obstetric emergencies as well as on maternal mortality. Various studies in India (34, 65, 81) report financial constraint and non-availability of human resources as the factors that delay the decision to seek delivery care, but these factors could not be captured in our study. We suggest that future research should embed the theoretical frameworks of delays and rights to capture these factors.

The trustworthiness of qualitative research depends on credibility (capturing the research questions well), dependability (taking the changes in the research process into consideration well), confirmability (grounding the interpretation well in the data), and transferability (applicability of the findings to other settings) (74). We took various measures to increase the trustworthiness of this study. We used the social autopsy method for data collection with structured and open-ended questions to address the overall research questions. This method is a methodological sub-field of verbal autopsy, and it has been used successfully in other qualitative studies on maternal and child deaths (64, 68). We triangulated the data collected from interviews with the data from various sources, and we triangulated the researchers having different expertise and experience levels. Two of the authors have been working in the study area in the capacity of programme officers of the United Nations Population Fund (UNFPA) for the last 6 years, working closely with the state government on strengthening the health care delivery services, including maternal health, whereas the other researchers were not familiar with the setting. The combination of researchers, with different expertise in the areas of medicine and social sciences and different levels of knowledge in the area and the issue under study, allowed both external and internal perspectives, which also added to the trustworthiness of the study (86).

## Conclusions

Our study revealed that women attempted to access maternal health care services when obstetric complications happened, but there were various socio-cultural and service delivery–related factors that delayed their access to appropriate care and contributed to maternal deaths in rural areas of Madhya Pradesh. We observed that the interconnectivity of different factors and delays reinforced and maintained the poor availability, accessibility, acceptability, and quality of services observed in the three phases. This study indicates that normative elements of a human rights approach to maternal health (i.e. the availability, accessibility, acceptability, and quality of maternal health services) were not upheld. The deceased women and their relatives were unable to claim their entitlements, and the duty bearers were not successful in meeting their obligations despite their conscious efforts to improve maternal health in the state. The data suggest that further concentrated efforts for better community education and health systems strengthening to provide appropriate and timely maternal health services, including EmOC, with good quality may positively affect the reduction of maternal mortality in this setting. The findings of our study indicated that further streamlined efforts are required for women's empowerment for enabling them in decision making. The other actions recommended are strengthening of the transportation and referral services, bringing positive change to the attitudes of maternal health care providers, and establishing appropriate accountability mechanisms.
